# IgG4-related disease—rare but you should not forget it

**DOI:** 10.1186/s42358-024-00374-y

**Published:** 2024-05-03

**Authors:** Frederico Augusto Gurgel Pinheiro, Ivanio Alves Pereira, Alexandre Wagner Silva de Souza, Henrique Ayres Mayrink Giardini, Rafael Alves Cordeiro

**Affiliations:** 1 Rheumatology Division, Universidade Federal de São Paulo (UNIFESP), São Paulo, SP Brazil; 2 Universidade do Sul de Santa Catarina (UNISUL), Florianopolis, SC Brazil; 3 Rheumatology Division, Faculdade de Medicina FMUSP, Universidade de Sao Paulo, Sao Paulo, SP Brazil; 4 Universidade Federal de São Paulo – Disciplina de Reumatologia, Rua Botucatu, 740, 3o andar, São Paulo, SP 04023-062 Brazil

**Keywords:** Immunoglobulin G4-related disease, IgG4-RD, Fibroinflammatory disease, Review, Rare diseases, Chronic relapsing-remitting inflammatory condition, Rheumatic diseases

## Abstract

Immunoglobulin G4-related disease is a systemic immune-mediated disease with insidious evolution characterized by fibroinflammatory lesions over virtually any organ system. Despite the remarkable progression of knowledge, its etiology remains undefined. Due to its relapse-remitting pattern, it could accumulate irreversible damage, increasing comorbidities and mortality. This paper emphasizes key concepts for diagnosing and treating patients with this condition.

## Introduction

Immunoglobulin G4-related disease (IgG4-RD) is a chronic fibroinflammatory disorder that was recognized in the 21st century and is characterized as a systemic and immune-mediated condition [1]. Other significant findings are abundant IgG4-positive plasma cells and fibroblasts [2]. Delaying adequate treatment results in organ dysfunction and even death.

IgG4-RD was first described as a specific entity in 2001. At this time, it was reported as a disease affecting specific organs, such as the pancreas and biliary tract, called sclerosing pancreatitis, and it was associated with elevated serum immunoglobulin G4 [3]. In 2003, still under the Japanese’s gaze, Kamisawa et al. described the systemic involvement in IgG4-RD [4].

The evolution of scientific knowledge has led to the inclusion of Kuttner tumor, Mikulicz disease, Ormond fibrosis, and Riedel thyroiditis in the IgG4-RD spectrum.

This review outlines relevant aspects of the epidemiology, pathophysiology, pathology, diagnosis, clinical manifestations, and treatment of IgG4-RD.

## Epidemiology

The global prevalence and incidence of IgG4-RD are still undetermined and underestimated, likely due to low awareness, a recent discovery, and an indolent clinical course [5, 6]. In a recent study of a claims-based analysis of commercially insured adults from the USA, the estimated incidence of IgG4-RD rose from 0.78 to 1.39 per 100,000 persons-years from 2015 to 2019 [7], which is similar to that published for granulomatosis with polyangiitis using a similar data source (∼1.0 per 100,000 persons) [8]. The estimated point prevalence for IgG4-RD on 1 January 2019 was 5.3/100,000 persons [7].

The typical patient with IgG4-RD is a middle-aged or older man, with a male-to-female ratio that ranges from 1.6:1 for head and neck involvement to 4:1 for other sites [6]. In Latin American patients with IgG4-RD, the mean age at the onset of symptoms and at the first visit were 48 and 50.8 years, respectively [9]. Despite the classic prevalence in the fifties to sixties [5], there are cases in the pediatric age group [10].

Current smoking was the first modifiable risk factor described for IgG4-RD, especially among those with retroperitoneal fibrosis [11]. In the occupational context of blue-collar workers, exposure to mineral dust, vapors, gases, fumes, and asbestos has been linked to an increased risk of developing IgG4-RD in the biliary tract and pancreas [12, 13].

According to Wallace et al. [7], patients with IgG4-RD have a 2.51 times higher risk of death than non-IgG4-RD patients (95% CI 1.76–3.56), warning about the relevance of early diagnosis and effective therapies.

## Pathophysiology

The pathophysiology of IgG4-RD is an affluent area that is continuously developing. Initially, the focus was on plasma cells due to high serum IgG4 levels [14]. However, other cell groups are currently recognized as core members of the pathophysiology. The activity in IgG4-RD has been linked to circulating and tissue B cells, plasmablasts, T follicular helper cells, T follicular regulatory cells, and cytotoxic T cells [5].

IgG4-RD has a biphasic evolution characterized by an initial inflammatory phase that can result in a final fibrotic process [6]. There are some descriptions of autoantigens (including galectin-3, annexin-A11, laminin-511, and prohibitin) that support the idea of triggers for the autoimmune response [6], but the precise mechanisms involved remain unclear [5]. The interaction between antigen-experienced B and T lymphocytes will result in the production of profibrotic molecules, such as interleukin 1β (IL-1β), interleukin 6, interferon γ (INF γ), transforming growth factor β (TGF β), platelet-derived growth factor β, and lysyl oxidase homologue 2 [6].

The most accepted theory for explaining the pathophysiology of IgG4-RD is that naïve B cells or plasmablasts present an antigen to a specific subset of T cells, the CD4 cytotoxic T lymphocytes (CTLs) [5, 6, 15, 16], resulting in tissue damage and fibrosis. These plasmablasts and CTLs express the signaling lymphocytic activation molecule F7 (SLAMF7; also CD319), a surface protein implicated in cell-cell interaction and chronic lymphocyte activation [6]. The CTLs often represent about 80% of all infiltrating CD4 T cells in tissues affected by IgG4-RD [16]. They are considered the significant drivers of tissue damage through the stimulation of the cytotoxic response (perforin and granzyme) and the production of profibrotic molecules (IL-1β, INF γ, TGF β) [6, 16–18].

Patients with IgG4-RD exhibit oligoclonal expansions of CTLs and plasmablasts that correlate with disease activity [5, 17], and the number of these cells is reduced after rituximab treatment [19, 20], which emphasizes the autoimmune etiopathogenesis of IgG4-RD. Another essential T cell subset is the CD4 follicular T helper (Tfh) cell, which drives the class switch to IgG4 (in plasma cells) [5, 6]. These observations suggest that the primary process responsible for the pathophysiology of IgG4-RD involves antigen presentation by B cells to CTLs [5].

IgG4 has been defined as a noninflammatory immunoglobulin in human physiology [21] that can be elevated in the context of chronic exposure to antigens (like in beekeepers), allergic diseases, and parasitosis [22]. The specific role of the IgG4 molecules in the pathophysiology of IgG4-RD has not been determined [5]. Still, it is known that serum IgG1 and IgG4 in patients with IgG4-RD leads to pancreatic and salivary damage in mouse models [23]. However, this model cannot be applied in humans since the human IgG4 has marked reduced affinity to the only inhibitory Fcγ in mice (FcγRIIB) [17]. Current research suggests that the elevations of IgG4 levels in IgG4-RD would be an effort to dampen aberrant immune activation rather than serve as the primary mediator of injury [5].

The final stage of the lesion is characterized by a dense stromal reaction, which can lead to organ distortion and sometimes irreversible damage [6]. The underlying mechanism includes the activation of fibroblasts by CTLs-derived mediators, resulting in excessive deposition of extracellular matrix proteins [17].

## Histopathology

**Fig. 1:**
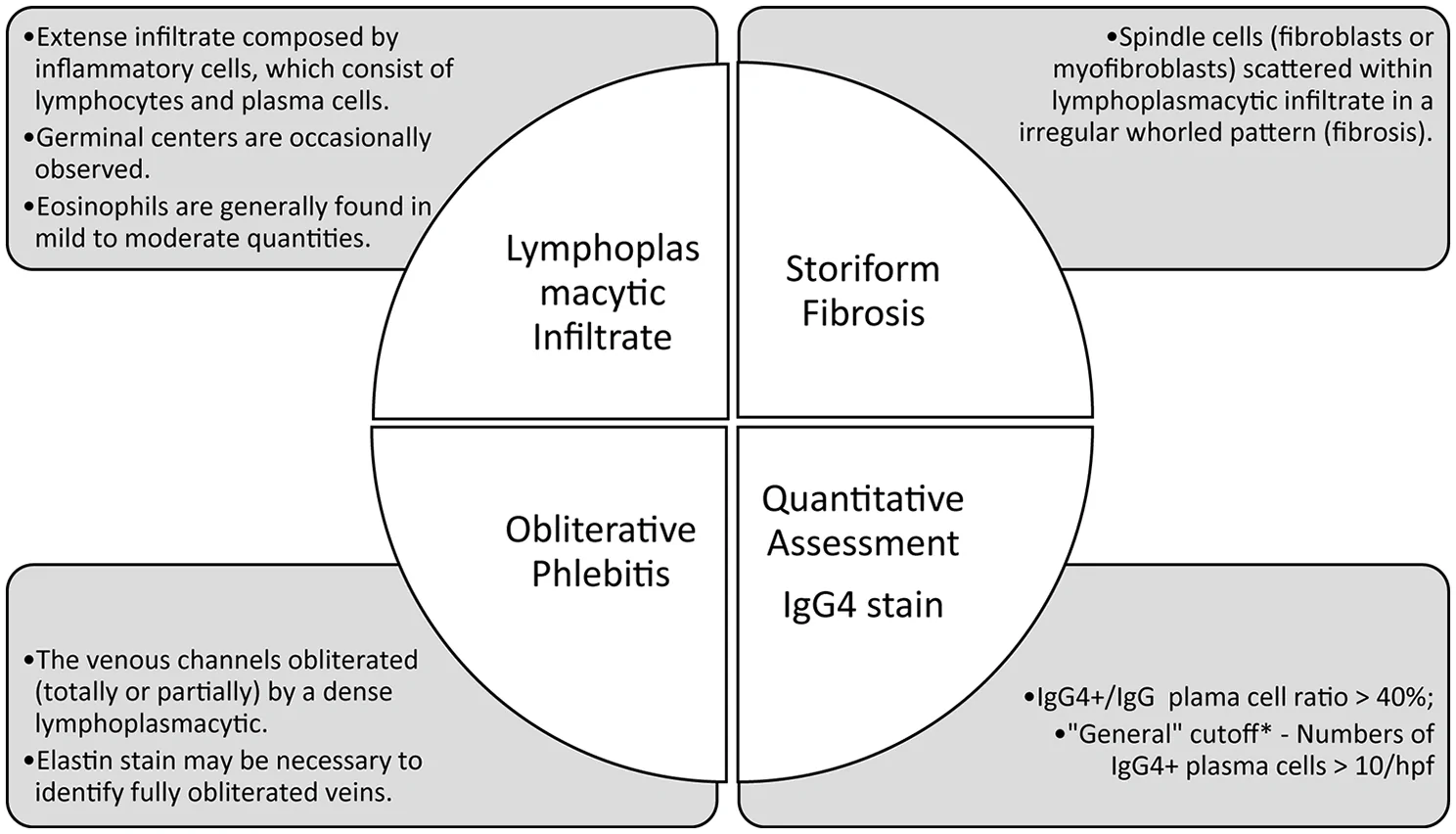
Histopathological features of IgG4-related disease. *The presence of > 10 IgG4+ plasma cells on biopsy specimens has been proposed as one component of a comprehensive diagnostic panel [25]. Still, the appropriate cutoff may vary from organ to organ, depending on the predominance of fibrosis at the time of the diagnosis [2]. *hpf* high-power field

The pathology is usually similar in the organs involved but with discrete singularities depending on the organ affected [24]. The classic histopathological findings of IgG4-RD are (Fig. 1): (a) dense lymphoplasmacytic infiltrate, (b) obliterative phlebitis, and (c) storiform fibrosis [2]. Other possible features are phlebitis without lumen obliteration or increased tissue eosinophils [2].

Obliterative phlebitis is described as venous channels totally or partially obliterated by a dense lymphoplasmacytic infiltration [2]. Arteritis can occasionally occur in specific organs (e.g., pulmonary lesions) [2]. Necrotizing lesions are not seen in IgG4-RD [2].

The storiform (derived from “storea”, a Latin word for woven mat [15]) fibrosis is composed of fibroblasts or myofibroblasts responsible for producing the fibrotic tissue arranged in an irregular whorled pattern [2]. Storiform fibrosis may not be detected with small needle biopsy samples [2].

Immunostaining (Fig. 1) is essential in analyzing the IgG4-RD lesions. Two characteristics are helpful for diagnosis: (a) a ratio of IgG4+/IgG plasma cells higher than 40% and (b) more than 10 IgG4 + plasma cells in the high-power field evaluation [2]. However, the appropriate cutoff point may vary depending on the organ (e.g., meninges generally have fewer IgG4 + plasma cells than lacrimal or salivary glands) and the size of the histopathological sample [2].

It is essential to understand that the five pathological hallmarks of IgG4-RD cannot define the diagnosis separately. The relative relevance can be perceived by the ACR/EULAR 2019 Classification Criteria for IgG4-RD when dense lymphocytic infiltrate punctuates “+4” (out of the 20 points necessary to the classification), and the association of dense lymphocytic infiltrate with storiform fibrosis punctuates “+13”, with or without obliterative phlebitis [26]. It is worth noting that even this “most specific” characteristic, storiform fibrosis, if present alone, can also occur in other diseases, such as dermatofibrosarcoma protuberans, benign fibrous histiocytoma [27], or Rosai-Dorfman disease [28]. Therefore, the axiom of the association of distinct data is necessary to diagnose IgG4-RD.

The presence of epithelioid cell granulomas or a prominent neutrophilic infiltrate typically suggests a diagnosis other than IgG4-RD [2, 24].

## Diagnosis

The accurate diagnosis of IgG4-RD depends on the combination of clinical, serological, radiological, and histopathological characteristics [15, 17]. Even relevant features (e.g., radiological characteristics) without pertinent association cannot define a diagnosis of IgG4-RD. ACR/EULAR adopted this concept to develop the 2019 classification criteria for IgG4-RD [26].

## Clinical manifestations

The clinical presentation is heterogeneous, and most patients have a chronic, indolent, and slowly progressive course [5, 17, 29, 30]. The signs and symptoms become evident over months or years [17, 29, 30], resulting in potentially irreversible damage-approximately 60% of IgG4-RD patients present damage at the baseline evaluation [31]. Fever is generally absent in IgG4-RD [5, 29]. Prominent fever (recurrent temperature > 38 °C) is pointed as an exclusion criterion by the 2019 ACR/EULAR classification criteria [26]. However, weight loss can occur, especially in patients with exocrine pancreatic dysfunction [5, 17].

Various studies frequently described allergic manifestations as important characteristics [1]. However, according to Mattoo et al. [32], the predominance of Th2 response was not observed in patients with IgG4-RD, except in those with previously known underlying atopic diathesis. Della-Torre et al. [33] showed that despite the perception that the prevalence of atopy was high among patients with IgG4-RD, the prevalence of atopy in their cohort was not different from that in the USA general population.

It has been observed that IgG4-RD affects almost all organ systems [1, 30]. However, it has preferences for specific organs: the major salivary glands (submandibular, parotid, and sublingual), the orbits and lacrimal glands, the meninges, the thyroid gland, the lungs, the pancreas and biliary tree, the aorta, the retroperitoneum, and the kidneys [26].

**Fig. 2:**
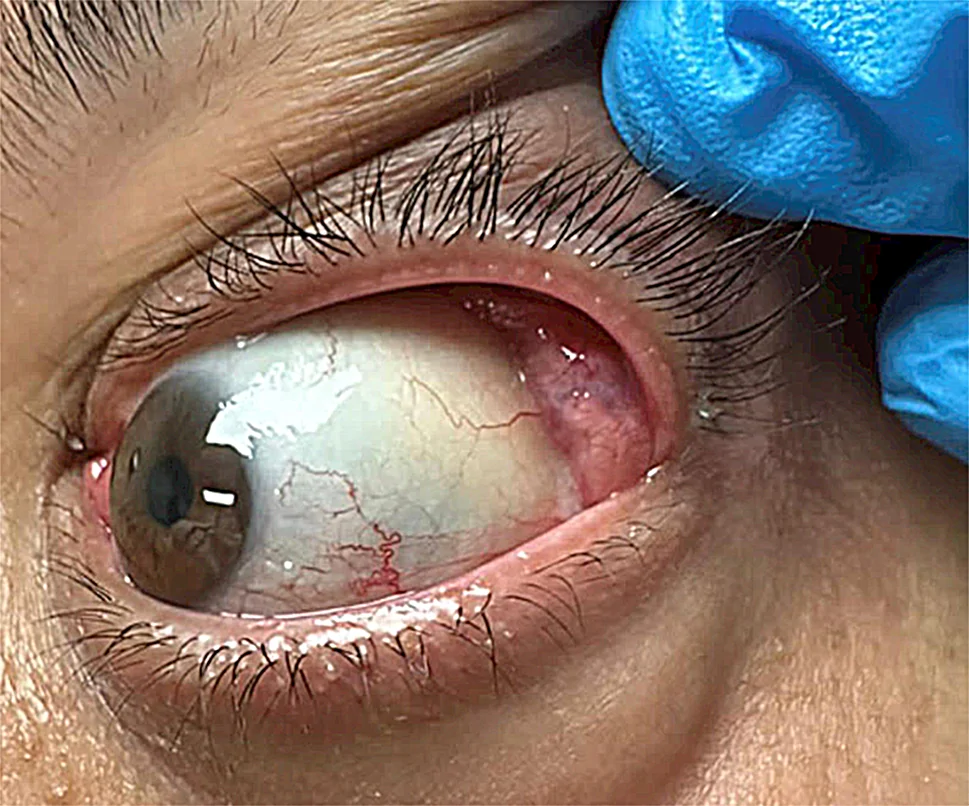
Lacrimal gland enlargement—evident after eversion of the eyelid. Courtesy of Dr. Rafael Alves Cordeiro and Dr. Henrique Ayres Mayrink Giardini

**Fig. 3:**
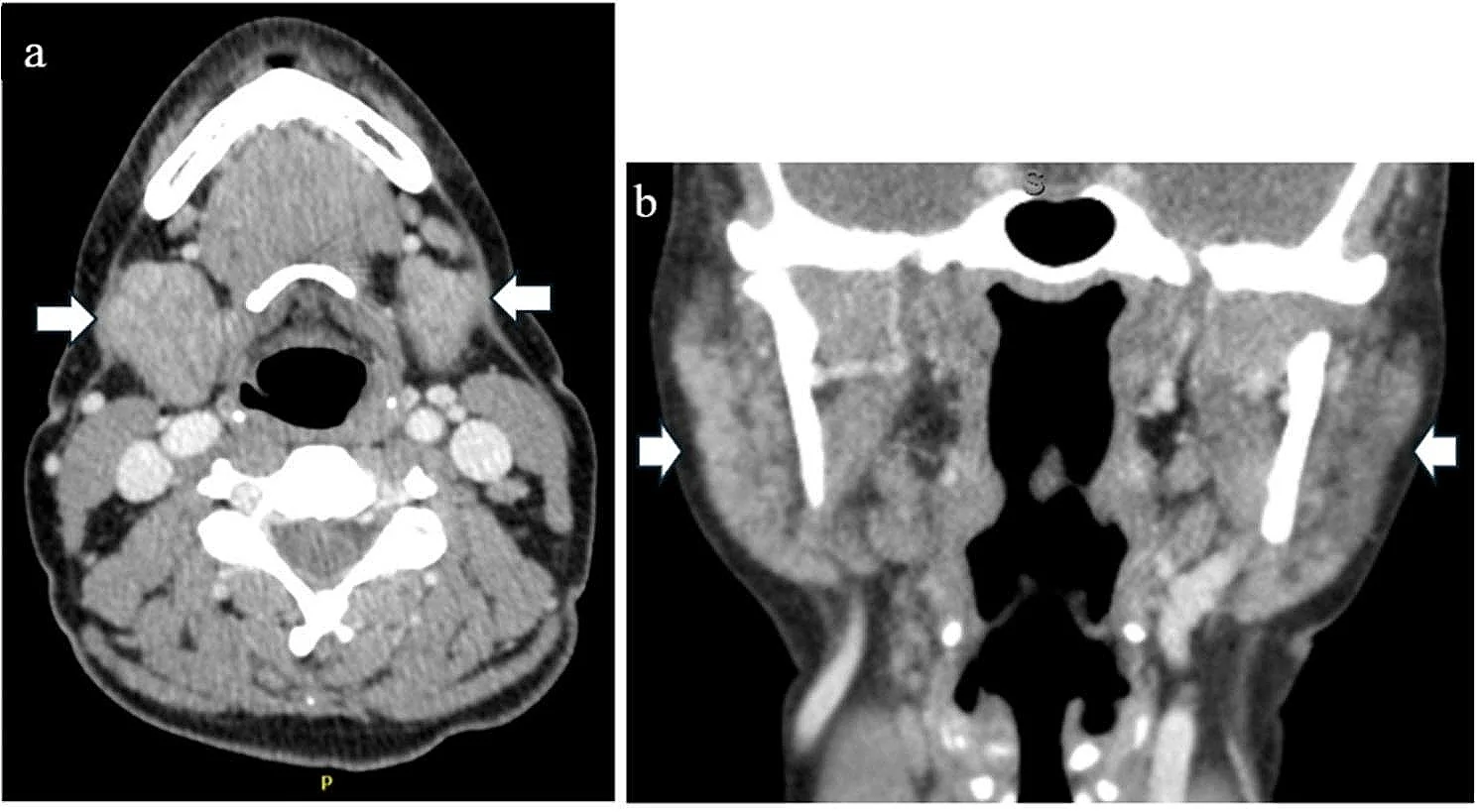
Representative radiological images of salivary gland lesions. (**a**) Submandibular gland enlargement (*white arrows*) of axial computed tomography (CT). (**b**) Parotid gland enlargement (*white arrows*) on coronal CT. Courtesy of Dr. Rafael Alves Cordeiro and Dr. Henrique Ayres Mayrink Giardini

**Fig. 4:**
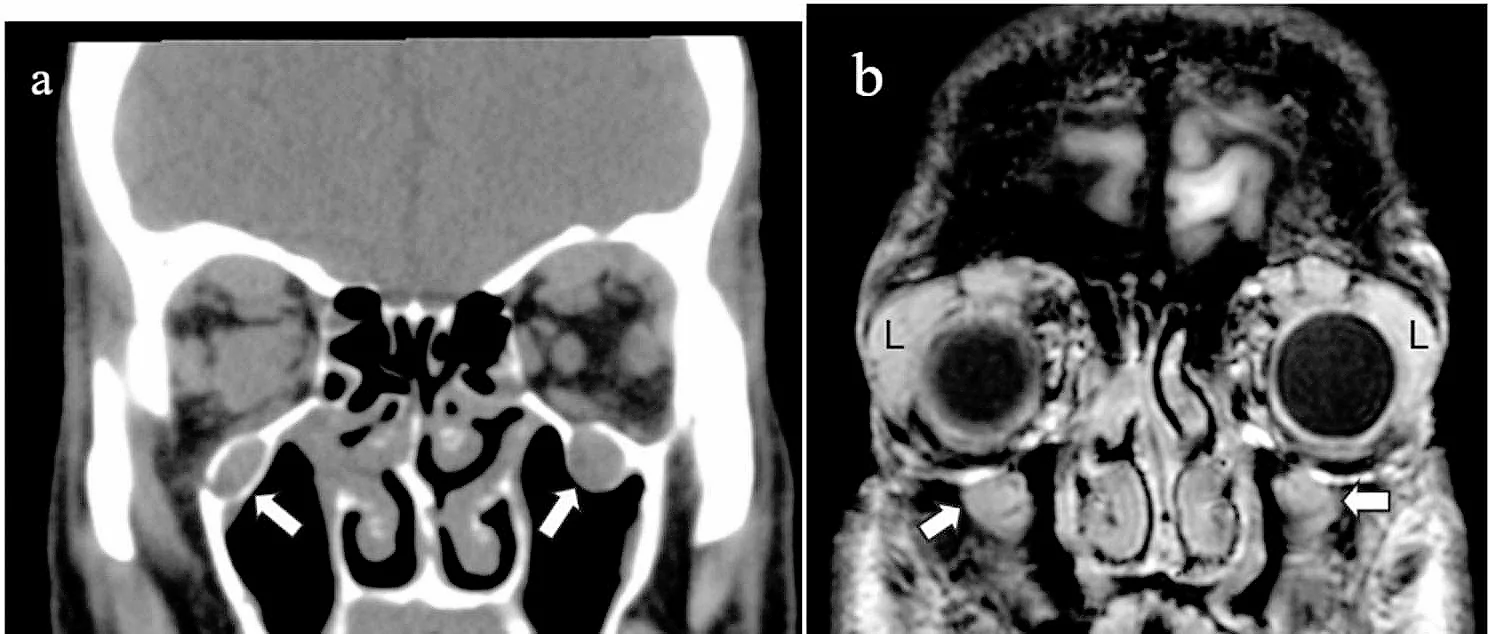
Radiological images of trigeminal nerve branch (infraorbital) and lacrimal lesions from the same case. (**a**) infraorbital nerve enlargement (*white arrows*) on coronal computed tomography; (**b**) lacrimal gland enlargement (*L*) and infraorbital nerve enlargement (*white arrow*) on a coronal gadolinium-enhanced T1-weighted image

**Fig. 5:**
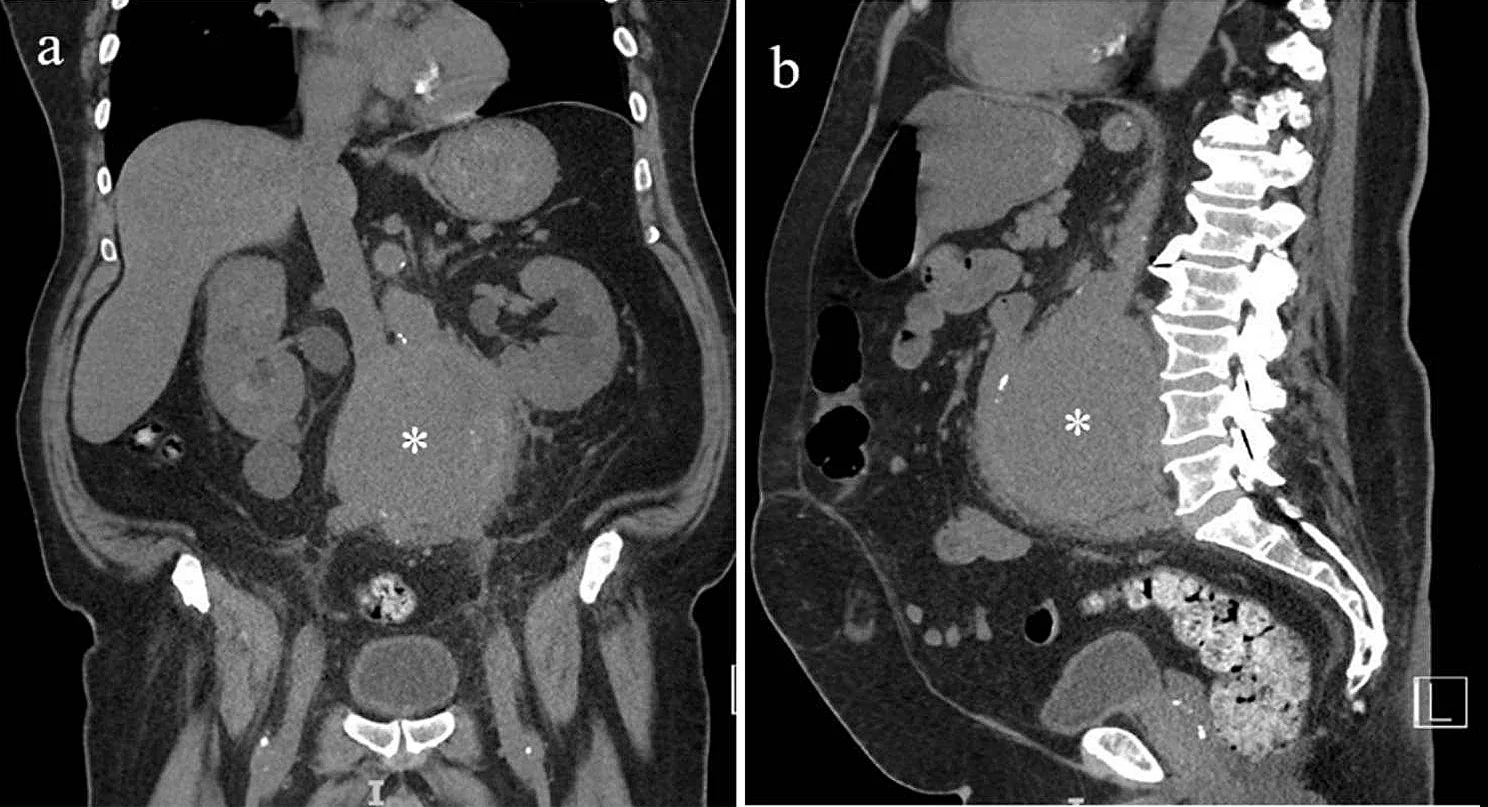
Radiological images of aortic involvement in the same patient. Pictures (**a**) and (**b**) on coronal and sagittal computed tomography revealed a large inflammatory aortic aneurysm (*). The pathologic evaluation revealed typical histopathological features of IgG4-related disease

Table 1; Figs. 2, 3, 4 and 5 list examples of manifestations and images observed in patients with IgG4-RD.

**Table 1:** The clinical spectrum of IgG4-related disease

Organ involvement	Manifestations	Clinical presentation/radiological or laboratory findings
**Head and neck**		
Eye and orbit	Dacryoadenitis Dacryocystitis Orbital myositis Orbital pseudotumor Scleritis (rare) Uveitis (rare)	Lacrimal gland enlargement (typically bilateral) Syndrome sicca Diplopia Proptosis Ptosis Red eye, ocular pain
Salivary glands	Sialadenitis of submandibular, parotid and/or sublingual	Glandular swelling Typically, bilateral Nontender or occasionally mild tender Syndrome sicca
Ear, nose, and throat	Chronic rhinosinusitis Midline destructive lesion Pharyngitis Supraglottic stenosis Vocal cord lesion	Allergic rhinitis symptoms, sinusitis, nasal polyps, rhinorrhea, nasal obstruction Nasal septum perforation (rare) Anosmia, middle ear effusion Hoarseness, Dyspnea
Thyroid gland	IgG4-related thyroiditis (Riedel)	Neck mass (woody) and pressure Hypothyroidism Recurrent laryngeal nerve palsy
**Thorax**		
Lungs and pleura	Parenchymal lung disease Pleural disease	Thickening of the bronco vascular bundles Interstitial lung disease, ground glass opacities Pulmonary nodules Pleural: effusion, thickening or nodules
Heart and pericardium	Coronary arteritis Pericarditis Pseudotumor	Perivasculitis around coronary arteries Coronary artery aneurysms Pericardial effusion Pericardial thickening Cardiac mass
Mediastinum	Fibrosing mediastinitis Paravertebral mass	Dyspnea, chest pain Mediastinal mass with compression of structures Paravertebral soft tissue, usually right-sided and located between T8-T11
Great vessels	Aortitis Periaortitis	Aortic wall thickening and enhancement Perivascular soft tissue around great vessels
Breast	IgG4-related mastitis	Painless breast mass
**Abdomen and pelvis**		
Pancreas	Autoimmune pancreatitis type 1 Pseudotumor	Obstructive jaundice, abdominal pain, diabetes mellitus, malabsorption Diffuse pancreatic enlargement (sausage-shaped pancreas) Capsule-like pancreatic rim Pancreatic atrophy (long-standing disease) Irregular narrowing of pancreatic duct Pancreatic mass (simulating adenocarcinoma)
Hepatobiliary system	Sclerosing cholangitis Sclerosing cholecystitis Hepatic inflammatory pseudotumor	Jaundice, weight loss, abdominal pain
		Mass mimicking cholangiocarcinoma
		Intra- and extrahepatic biliary ductal dilatation
		Gallbladder wall thickening
		Hepatic mass, transaminitis
Kidneys	Tubulointerstitial nephritis Membranous glomerulonephritis	Elevated creatinine, proteinuria, hematuria, nephritic/nephrotic syndrome Diffuse kidney enlargement Renal cortical hypodensities Renal pelvic wall thickening Renal atrophy (long-standing disease)
Retroperitoneum	Retroperitoneal fibrosis (Ormond’s disease) Aortitis and periaortitis	Back/flank pain, leg edema, hydronephrosis, deep venous thrombosis, varicocele Retroperitoneal mass or fat stranding Periaortic inflammation involving all or parts of the aorta distal to the renal arteries, extending to the iliac arteries
**Nervous system**		
Pituitary	Hypophysitis	Hypopituitarism, diabetes insipidus, headache Pituitary enlargement Thickened pituitary stalk
Meninges	Hypertrophic pachymeningitis	Headache, cranial nerve palsies, vision disturbance, sensorineural hearing loss, seizures, motor weakness, limb numbness Hypertrophic and nodular meningeal thickening Hydrocephalus
Central nervous system		Dementia, hemiparesis, multifocal neurological defects
Peripheral nerves	Often asymptomatic—incidental radiological finding	Perineuritis, most typically of the infraorbital nerve
**Lymph nodes**	Often asymptomatic	Lymph node enlargement (generalized or localized), generally 1–3 cm in diameter, non-tender Does not have a preference for any set of lymph node

The table was adapted from multiple Refs. [5, 29]

## Clinical phenotypes

The international cohort of patients with IgG4-RD identified four phenotypes: Group 1—pancreatohepatobiliary (31%), Group 2—retroperitoneum and aorta (24%), Group 3—head and neck limited disease (24%), and Group 4—classic Mikulicz’s syndrome and systemic (22%) [34]. These categorizations allowed the identification of unique findings: Group 1 (pancreatohepatobiliary) had more urgent manifestations (admission to the emergency department, generally due jaundice or abdominal pain) of IgG4-RD [35] than other groups; patients in Group 3 (head and neck limited) had more youngers, females and Asian individuals than the other groups; Group 4 (Mikulicz’s syndrome and systemic involvement) had the highest levels of serum IgG4 levels [34]. However, there is a significant overlap among organ system involvement and phenotypes in this classification [16].

## Differential diagnosis

The differential diagnosis should consider the affected organ system. Some diseases, such as sarcoidosis, granulomatosis with polyangiitis, Erdheim-Chester disease, Castleman disease, and Rosai-Dorfman disease, have similar presentations and must be considered for most patients with IgG4-RD [5]. Another important group of diseases is malignancies (hematological and solid tumors).

Analyzing the exclusion criteria presented in the 2019 ACR/EULAR classification criteria is a valuable tool in the workup of IgG4-RD and mimickers [26]. Grouped relevant features into aspects to exclude IgG4-RD [25]:

- *clinical features*: fever or no objective response to glucocorticoids;
- *serologic evaluation*: the positive result of Enzyme-linked immunosorbent assay for antineutrophil cytoplasmic antibody (ANCA) against proteinase 3 or myeloperoxidase, the presence of specific autoantibodies (such Anti-Ro, -La, -DNA, or others specific), peripheral eosinophilia > 3000 mm^3^, cryoglobulinemia, or leukopenia and thrombocytopenia without alternative explanation;
- *radiologic features*: the presence of radiologic findings that suggest malignancy or infection, rapid radiologic progression (worsening within 4–6-week intervals), long bone abnormalities consistent with Erdheim-Chester disease or splenomegaly (>14 cm in the absence of alternative explanation);
- *pathologic evaluation*: cellular infiltrates suspicious for malignancy, prominent neutrophilic inflammation, presence of fibrinoid necrosis, prominent necrosis, inflammation rich in epithelioid histiocytes (granulomatous inflammation), markers consistent with inflammatory myofibroblastic tumor or pathologic features of macrophage/histiocytic disorder.

## Biomarkers

In patients with IgG4-RD, the first biomarker remembered is the serum IgG4 concentration. Measuring IgG4 levels is helpful, especially for patients with elevated IgG4 levels at diagnosis [6]. However, the serum IgG4 concentration is not a perfect biomarker since (a) it is not specific for IgG4-RD (many conditions can be associated with elevated serum IgG4 levels, including infectious diseases) [36]; (b) it may be normal or slightly elevated in specific subtypes of patients with IgG4-RD, such as those with retroperitoneum and aorta phenotype (Group 2) [34]; (c) serial measurement of serum IgG4 may not be completely reliable (up to 63% of cases, serum IgG4 levels do not normalize after glucocorticoid treatment; and in approximately 10% of cases, IgG4 levels do not increase again during disease flare) [37].

Table 2 shows examples of IgG4-RD’s biomarkers for diagnosis and follow-up.

**Table 2:** Biomarkers of IgG4-related disease

Type of biomarker	Examples	Comments
Diagnosis	Serum IgG4	Elevated in 55–97% of patients. Correlates with disease burden
	Serum IgG4/IgG ratio	When > 10%, it increases specificity in the case of normal serum IgG4
	Serum IgE and eosinophils	Elevated in 30% of patients regardless of atopic background
	CSF IgG4 indices	High in IgG4-related hypertrophic pachymeningitis
	Plasmablasts and plasma cells	Expanded in peripheral blood regardless of serum IgG4 concentration
	Serum C3 and C4	Reduced levels suggest active disease, particularly in patients with IgG4-related kidney disease (mostly tubulointerstitial nephritis)
	CD4 and/or CD8 SLAMF7 + CTLs	Expanded in peripheral blood during active disease
	^18^FDG-PET	Useful for staging purposes and the definition of alternative biopsy sites. Caution when interpreting lymph nodes (indistinguishable from reactive and neoplastic lymph nodes)
Disease activity	Serum IgG4, IgE, and eosinophils	Decrease with disease response to treatment. It may not normalize at disease remission. Marked (> 2x) increase after remission should raise the possibility of disease flare
	Serum IgG4/IgG ratio	Decrease in disease response to treatment
	CSF IgG4 indices	Decrease in disease response to treatment
	Plasmablasts and plasma cells	Decrease in disease response to treatment and increase at flare
	Serum C3 and C4	They may normalize in remission and decrease during flares, especially in kidney involvement
	Serum ESR/CRP*	Correlate with disease activity, especially in retroperitoneal and aortic involvement
	CD4 and/or CD8 SLAMF7 + CTLs	Decrease with disease response to treatment and increase with flare
	^18^FDG-PET	^18^FDG uptake reduced after treatment. Caution when interpreting lymph nodes (indistinguishable from reactive and neoplastic lymph nodes)

*CRP* C-reactive protein, *CSF* cerebrospinal fluid, *CTL* CD4 cytotoxic T lymphocyte, *ESR* erythrocyte sedimentation rate, *FDG* fluorodeoxyglucose, *PET* positron emission tomography

*The elevation is higher for ESR than in CRP. Elevated CRP levels tend to be mild. Adapted from Refs. [5, 6]

## Treatment

IgG4-RD is a treatable disease, but the necessity of treatment depends on the organ involved, the risk to the organ, or the life-threatening. The involvement of the pancreas, meninges, aorta, or kidneys is associated with a poor prognosis, indicating the need for early treatment [38]. However, most experts agree that asymptomatic disease limited to a single organ, with a minimal amount of tumor/mass, and with a low risk of progressing to long-term organ dysfunction may not require treatment [38]. Asymptomatic lymphadenopathy or mild submandibular enlargement are examples of IgG4-RD in which treatment might be deferred (“watchful waiting” strategy) [16, 38, 39].

Another essential premise for defining the most appropriate treatment is a complete evaluation of comorbidities, disease extension, and the presence of risk factors for relapse [38].

The risk factors for relapse are male sex, the number of organs involved at baseline, higher concentrations of serum IgG4, serum IgE or eosinophils at baseline [16], and hypocomplementemia [5].

After induction therapy, maintenance treatment is indicated for patients with risk factors for relapses at disease onset [38].

**Fig. 6:**
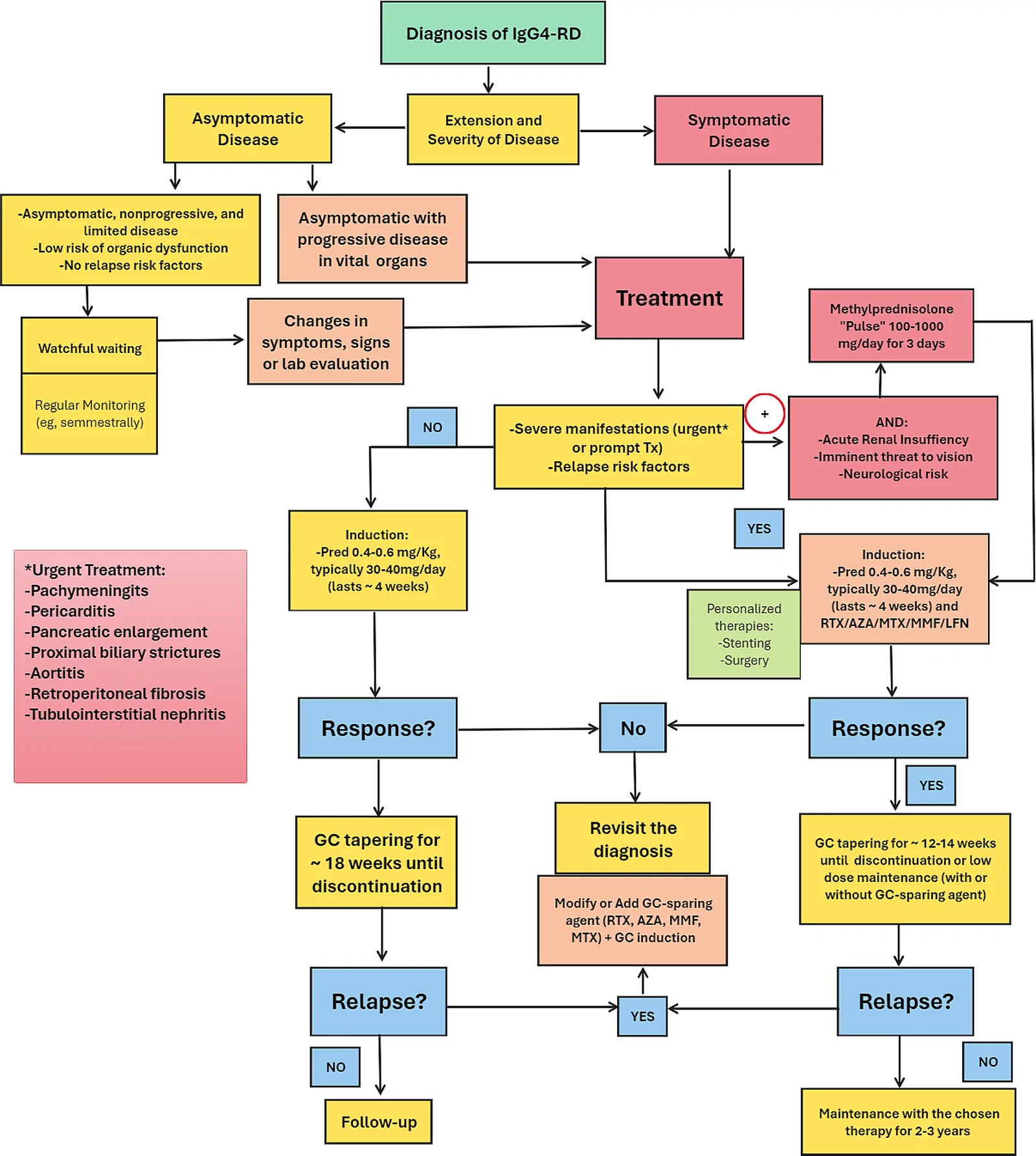
A practical approach to treating patients with IgG4-related disease. *AZA* azathioprine, *GC* glucocorticoids, *LFN* leflunomide, *IgG4-RD* IgG4-related disease, *MMF* mycophenolate mofetil, *MTX* methotrexate, *Pred* prednisone, *RTX* rituximab, *Tx* treatment, ∼ approximately. This figure was adapted from multiple Refs. [14, 38, 39]

A practical approach for treating patients with IgG4-RD is presented in Fig. 6.

Glucocorticoids (GC) are the cornerstone for remission induction in all patients with active disease unless contraindications exist [16]. Patients with IgG4-RD typically respond well to GC, but if an inadequate result is observed, the diagnosis should be reevaluated according to the ACR/EULAR classification criteria [26]. For the induction of remission, Wu et al. [40] showed that high (0.8-1 mg/Kg/day) vs. medium (0.4–0.6 mg/Kg/day) doses had similar effects. Medium doses of 30–40 mg/day are preferred for most patients, while higher doses are reserved for critical cases [38]. This initial chosen dose of GC must be maintained for 4 weeks and gradually tapered down (e.g., by 5 mg every 2 weeks) until a maintenance dose ≤ 7.5 mg/day is reached [38]. The duration of GC induction and maintenance treatment may be adjusted depending on the presence or the risk of developing GC-related side effects [38]. The speed of GC should be individualized, but faster decreases are related to an increased chance of relapses [38]. The duration of the GC induction therapy tends to be 3–6 months in most centers [38]. Regarding GC maintenance, there is a tendency for Asian countries to use prednisone in low doses (5–10 mg/day) for up to 3 years. However, it is more common in Western countries to use prednisone for a short period, with prolonged periods only for specific cases (refractory disease or recurrence shortly after prednisone cessation) [16].

There is a tendency for experts to consider the use of GC in combination with conventional disease-modifying anti-rheumatic drugs for IgG4-RD as a steroid-sparing strategy. However, only small observational or retrospective studies were conducted to evaluate the use of these combinations [38]. The commonly associated drugs with GC are azathioprine, mycophenolate mofetil, methotrexate, leflunomide, and cyclophosphamide [16].

Among the biological agents, the rituximab (anti-CD20; two intravenous 1000 mg doses, administered 15 days apart) demonstrated efficacy in a prospective open trial of 30 patients with IgG4-RD, with disease response occurring in 29 (97%) patients [41]. In this study, 23 (77%) patients achieved the primary outcome of remission, and 12 (40%) patients achieved complete remission at 12 months [41]. As a maintenance therapy, 1000 mg of rituximab was administered semiannually to prevent IgG4-RD flares in patients at risk of relapses, as data from a small study indicated [42].

Other biological agents are being studied: (a) obexelimab, a humanized monoclonal antibody that binds CD19 and Fc gamma receptor IIb to inhibit B lineage cells, showed promising results in a phase 2 trial recently published [43]; a phase 3 study is ongoing (clinical trials.gov: NCT05662241); (b) A phase 3 study with inebilizumab, a humanized IgG1 kappa monoclonal antibody against CD19 (clinical trials.gov: NCT04540497); (c) elotuzumab, a monoclonal antibody directed against SLAMF7 (clinical trials.gov: NCT04918147). Other therapies that act through interactions between B and T cells and their stimuli (such as those mediated by T follicular helper) are under evaluation, providing a good perspective for patients and physicians.

Surgical treatment or the apposition of medical devices (stents) are complementary approaches for specific situations [39]. Occasionally, surgery can be necessary for severely damaged organs. Nephrostomy tubes, biliary, or ureteral stents may be required initially but should be removed promptly to prevent complications [38].

## Conclusion

IgG4-RD is a new entity recognized in the 21st century. Patients with this disease have a broad range of manifestations, which should be considered in the differential diagnosis of many conditions. Whether adequately conducted, it spares a relevant burden of comorbidities and mortality. Distinct specialties can be consulted, and knowledge about IgG4-RD can allow for prompt recognition and the institution of corrected treatment, reducing the risk of progressive damage.

## Data Availability

Data sharing does not apply to this article as no datasets were generated or analyzed during the current study.

## Abbreviations

ACR: American college of rheumatologists
ANCA: Antineutrophil cytoplasmic antibody
CI: Confidence interval
CRP: C-reactive protein
CSF: Cerebrospinal fluid
CTLs: CD4 cytotoxic T lymphocytes
ESR: Erythrocyte sedimentation rate
EULAR: European league against rheumatism
FDG: Fluorodeoxyglucose
HPF: High-power field
IgG4: Immunoglobulin G4
IgG4-RD: Immunoglobulin G4-related disease
IL-1β: Interleukin 1β
INFγ: Interferon γ
PET: Positron emission tomography
SLAMF7: Signaling lymphocytic activation molecule F7
TGF-β: Transforming growth factor β
USA: United States of America
